# Ten strategies to optimize early mobilization and rehabilitation in intensive care

**DOI:** 10.1186/s13054-021-03741-z

**Published:** 2021-09-03

**Authors:** Carol L. Hodgson, Stefan J. Schaller, Peter Nydahl, Karina Tavares Timenetsky, Dale M. Needham

**Affiliations:** 1 Australian and New Zealand Intensive Care Research Centre, School of Public Health and Preventive Medicine, Monash University, 3/553 St Kilda Rd, Melbourne, VIC 3004 Australia; 2 Department of Intensive Care and Hyperbaric Medicine, The Alfred, Melbourne, VIC Australia; 3 Charité – Universitätsmedizin Berlin, corporate member of Freie Universität Berlin, Berlin, Germany; 4 Department of Anesthesiology and Operative Intensive Care Medicine, Humboldt-Universität zu Berlin, Chariteplatz 1, Berlin, Germany; 5 Department of Anesthesiology and Intensive Care, School of Medicine, Technical University of Munich, Munich, Germany; 6 Nursing Research, Department of Anaesthesiology and Intensive Care Medicine, University Hospital of Schleswig-Holstein, Kiel, Germany; 7 Department of Critical Care, Hospital Israelita Albert Einstein, São Paulo, SP Brazil; 8 Outcomes After Critical Illness and Surgery (OACIS) Group, Division of Pulmonary and Critical Care Medicine, Department of Physical Medicine and Rehabilitation, School of Medicine, Johns Hopkins University, Baltimore, MD USA; 9 School of Nursing, Johns Hopkins University, Baltimore, MD USA

## Introduction

In the last decade, there have been more than 40 randomized trials evaluating early mobilization and rehabilitation in intensive care units (ICU) [1]. Such trials generally aim to reduce the incidence of ICU-acquired weakness (ICUAW) which is associated with poor long-term survival, physical functioning, and quality of life [2]. At least eight international guidelines have recommended ICU early mobilization and rehabilitation [3].

Despite supporting evidence and guidelines, implementation of ICU mobilization and rehabilitation is highly variable[4]. Hence, we report on 10 steps to help ICU clinicians in optimizing early mobilization and rehabilitation.

## Create multidisciplinary team with designated champions

Early mobilization and rehabilitation is more successful in ICUs with a culture that prioritizes and values this intervention [5]. Mobility champions can help develop this culture using leadership and communication skills to educate, train, coordinate, and promote patient mobilization [3, 4, 6]. They support staff with an emphasis on safety and practical skills to improve the team’s confidence and capabilities [6].

## Use structured quality improvement (QI) processes

A structured QI approach can greatly enhance successful implementation of early mobilization and rehabilitation [7]. One approach to QI includes four steps: (1) summarizing the evidence; (2) identifying barriers (e.g., sedation or lack of equipment); (3) establishing performance measures (e.g., sedation targets, frequency, and level of patient mobilization); and (4) ensuring all eligible patients receive the intervention (via appropriate engagement, education, execution, and evaluation) [6, 7].

## Identify barriers and facilitators

A systematic review identified 28 unique barriers to early mobilization and rehabilitation, including patient-related barriers (e.g., physiological instability and medical devices), structural barriers (e.g., limited staff and equipment), procedural barriers (e.g., lack of coordination and delayed screening for eligibility), and cultural barriers (e.g., prior staff experience and ICU priorities for patient care) [4]. There are many strategies to effectively overcome barriers, including implementation of safety guidelines; use of mobility protocols; interprofessional training, education, and rounds; and inclusion of physician champions [4].

## Promote multi-professional communication

The multi-professional team effort required for early mobilization and rehabilitation program depends on optimal communication. We recommend that interprofessional communication is facilitated using a structure adapted to the individual ICU that allows (algorithm-based) mobilization goals, including an opportunity for all team members to raise concerns and ensure flow of information regarding mobility goals and achievement across staff and over time [8].

## Understand patient preferences

**Fig. 1:**
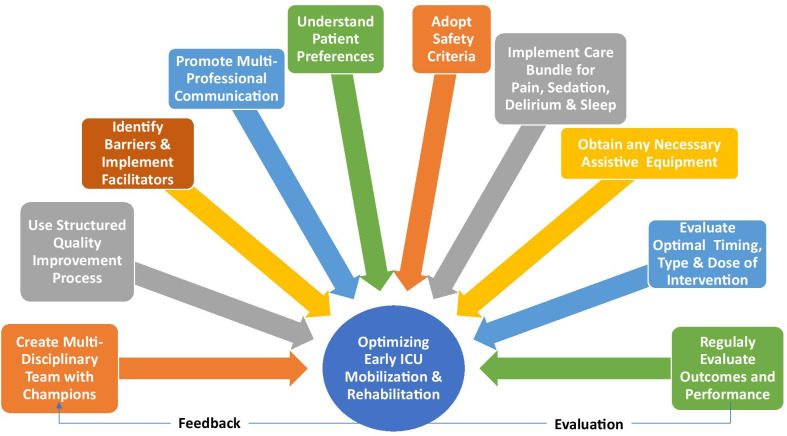
Ten strategies to optimize early mobilization and rehabilitation in ICU

ICU patients’ experience with early mobilization and rehabilitation is variable. It may be tiring, uncomfortable and difficult, while at other times be motivating and rewarding for patients [9]. With improving cognitive status, patients may be shocked by the severity of their muscle weakness. In the early stages of critical illness, patients may prefer to focus on short-time goals (e.g., sitting in a chair) set by the multidisciplinary team [9]. As patients progress, they may become more engaged in goal setting and longer-term rehabilitation planning (e.g., walking longer distances, sitting outside) (Fig. 1).

## Adopt safety criteria

Meta-analyses have demonstrated the safety of in-bed and out-of-bed ICU mobilization, with rare occurrence of serious events [10]. One method of assessing safety is a traffic light system that provides specific criteria, across respiratory, hemodynamic, neurological, and other body systems, to be considered in mobilizing individual patients [11]. In this system, “red light” criteria indicate an increased potential for a serious safety event during mobilization requiring experienced decision-making, “yellow light” indicates potential risk that should be evaluated in terms of benefits versus risks, and “green light” indicates that mobilization is generally safe [11].

## Implement care bundles for pain, sedation, delirium, and sleep

Patients’ sedation and delirium status is a common barrier to early mobilization and rehabilitation [4]. More broadly, pain, sedation, delirium, sleep, and early mobilization and rehabilitation are closely inter-related, as considered in clinical guidelines[3]. Assessment and management of these issues, via existing evidence-based practices (as synthesized in the guidelines), are important to maximize patients’ ability to participate in rehabilitation.

## Obtain any necessary equipment

Barriers to early mobilization and rehabilitation may include ICUAW, impaired physical functioning, traumatic injuries, and obesity [6]. Equipment can expand treatment options, increase patient mobility and activity levels, and reduce risk of injury to staff [12]. Selecting rehabilitation equipment may be challenging, with important considerations including the equipment cost/availability, ability to share equipment between units or patients (including infection control considerations), and the physical space available for patient mobilization and for convenient storage of equipment. Evidence supporting use of specific equipment is still evolving, including evaluation of neuromuscular electrical stimulation (NMES), in-bed cycle ergometry, tilt tables, and other devices [12, 13].

## Evaluate optimal timing, type, and dose of intervention

Important knowledge gaps exist regarding exercise, including the timing, type, and dose of interventions. There is some evidence suggesting that starting rehabilitation within 2 or 3 days of ICU admission may be superior to later initiation [3]. Types of interventions to be considered include active functional mobilization, in-bed cycle ergometry, electrical muscle stimulation (with or without passive/active exercises), tilt tables, and use of various rehabilitation equipment. In addition, the intensity, duration, and frequency of each intervention type are important considerations [14]. Additional research is needed to further understand potential benefit or harm. Until that time, clinician judgement will play an important role and must be tailored to individual patients and to the dynamic nature of critical illness.

## Assess outcomes and performance

Mobility and rehabilitation-related measures, appropriate to the ICU setting and integrated into clinical care, are needed to set patient goals and track their progress, allocate scarce rehabilitation resources to those patients who may benefit the most, and conduct evaluations of structured quality improvement programs [15]. Understanding patients’ functioning prior to critical illness, and their own goals, are also important considerations.

## Conclusion

Evidence is still evolving about early mobilization in ICU with ongoing large, multi-center trials. Further research is needed to understand the optimal timing, type and dose of interventions, and their effect on long-term patient outcomes. These 10 strategies provide guidance for implementing early mobilization and rehabilitation in the ICU with the goal of optimizing safety and effectiveness to improve patients’ experiences and outcomes.

## Data Availability

Not applicable.
